# Transactions of the Fourth Annual Meeting of the American Association of Obstetricians and Gynecologists

**Published:** 1892-03

**Authors:** 


[*From The American Journal of Obstetrics.*]

### TRANSACTIONS OF THE FOURTH ANNUAL MEETING OF THE AMERICAN ASSOCIATION OF OBSTETRICIANS AND GYNECOLOGISTS.

---

HELD IN NEW YORK CITY, SEPTEMBER 17, 18, AND 19, 1891, AT THE ACADEMY OF MEDICINE.

---

(*Abstract.*)

### THIRD DAY—MORNING SESSION.

THE President, Dr. A. H. WRIGHT, of Toronto, in the chair.

Discussion of papers by Dr. Wathen and Dr. Marcy, resumed.

Dr. W. W. POTTER, of Buffalo, said he had kept watch of Dr. Marcy's work in this direction, and had become somewhat familiar with his writings on the subject. His little treatise, upon hernia, published two years ago, was a revelation to many with reference to the modern technique of the operations for the several forms of hernia. Dr. Potter reported a case of ventral hernia upon which he had operated fourteen years ago with success.

Dr. R. B. Hall, of Cincinnati, said he hoped that Dr. Marcy, in closing, would give in detail the method of closing the abdominal wound after operating for hernia—whether he advises and uses the kangaroo tendon in closing the peritoneum. He had operated a number of times, using catgut, and was disappointed in its results.

Dr. J. H. Carstens, of Detroit, illustrated on the board the old-fashioned method of including all the tissues in one sweep, resulting frequently in hernia, because too much of the peritoneum was included in the tissues taken up. He said that if you will take catgut or kangaroo tendon, or whatever you like, and sew, layer by layer, up to the skin, you will get perfect union; you get peritoneum together, you get fascia together, and you get no umbilical hernia. If you follow in the track of Lawson Tait, you will be astonished at the number of cases of umbilical hernia you will find; and if Mr. Tait would advance to the American plan, and accept a little from us, and make use of the buried animal suture, he would very seldom have a case of hernia following an operation.

Dr. R. T. Morris, of New York, wished to lay emphasis upon the danger of peritoneal hernia where the operation was performed with one suture, not bringing the different layers together successively.

Dr. Jas. F. W. Ross, of Toronto, said he regretted that we were not able at the present moment to decide the question between the method of taking peritoneum, skin and fascia together, and the other method of suturing each separately. He had always adopted the single suture, taking up all the tissues, and up to the present time had only two cases of hernia. He wished to ask Dr. Marcy whether it had been experimentally proved that fibrous tissue unites to fibrous tissue, and can be traced absolutely as fibre in the scar; whether muscle unites to muscle, and can be traced as muscular tissue in the scar. On the surface, where we can see, we find it is not true skin in the scar.

Dr. H. O. Marcy, closing the discussion, said he wished to speak more particularly in reference to the subject presented by Dr. Wathen. Dr. Wathen's excellent results showed him to be a very careful operator, but there were some points in which he did not agree with his method of operating. It is some twenty years since, as a pupil of Mr. Lister, Dr. Marcy first began the study of wound treatment. During this time he had carried on many careful bacteriological experiments in his own laboratory. He thought it would not be quite right to accept Dr. Wathen's conclusion that it is no longer antiseptic surgery, but aseptic surgery, although the aseptic wound is our highest ideal and is that to which we strive to bring the result.

We place far too much emphasis on what we call the atmospheric conditions, and far too little on a dirty finger nail. He was not prepared to feel that he could afford to dispense with irrigation. Another point was, not to fill the wound with a dozen or twenty compression forceps, and then expect union by first intention.

In his last hundred laparatomies he had turned on an aseptic gas—oxygen gas—the moment he entered the abdominal cavity. It is a non-irritating gas, and displaces the column of infected air that would otherwise gain access to the wound.

Septic wounds are to be treated in one way, aseptic wounds in another; the latter should never have a drainage-tube applied. He uses a far less number of drainage-tubes than formerly, and insists upon it that the drainage-tubes shall be smaller, shorter, and less in number.

In reference to sutures, silk is encysted; it is not absorbed. He had removed it after it had been in place three years. Silk-worm gut is equivalent to wire. At the best the tissues encapsulate it; they do not utilize it by absorption or a replacement of other tissues. For many years he was satisfied with catgut; he prepared his own; but he was brought to feel that catgut was not reliable—may not be reliable. The best tendons which he had been able to find were those taken from the tail of the Australian kangaroo, after experimenting with tendons from the deer, moose, squirrel, caribou, buffalo and other animals.

He illustrated on the board the anatomical conditions pertaining to umbilical hernia.

Dr. X. O. Werder, of Pittsburg, Pa., reported cases illustrating

### SOME MOOT POINTS IN ECTOPIC GESTATION.

On the 23d of May last he operated on a patient, thirty-eight years of age, mother of five children, in whom the menses had been regular up to six weeks previous to operation, during which time there had been a constant flow of blood. A mass in Douglas's cul-de-sac had been made out, pushing the uterus to left of median line, soft, semi-fluctuating, of the size of a large orange. After intestines and omentum which were adherent to this mass had been separated, a blood tumor was found, and several handfuls of blood coagula having been emptied out of the abdominal cavity, the ruptured, dilated right tube, containing a placenta, was brought up into the abdominal incision and ligated. The abdominal cavity had been perfectly free from blood previous to breaking into this mass, and there were no signs of any inflammatory trouble. There was no doubt that this tumor was a pelvic hematocele, caused by tubal pregnancy rupturing into the abdominal cavity. Rupture had undoubtedly occurred, as the history shows, about six weeks previous to operation.

This case illustrates, contrary to the teachings of Tait and others, that not all cases of intraperitoneal ruptures of tubal pregnancy prove fatal.

Two other cases seen by the author, of undoubted ruptured tubal pregnancy into the abdominal cavity, in which operation, urged as the only hope of recovery, was refused, also recovered, though convalescence was slow and tedious; in fact, the patients, though months have elapsed since occurrence of this intraperitoneal hemorrhage, are still more or less invalids.

In two other cases laparatomy was not performed until five days after rupture had taken place, when the patients were recovering from their condition of collapse and apparently improving. Out of a total of six cases of tubal pregnancy, therefore, in five recovery would have been probable without operation.

The author therefore concludes:

1. Intraperitoneal rupture is not always fatal; a large percentage recover without operation.

2. The convalescence of those recovering without operation, in his experience, is very slow and tedious, and often, perhaps, impossible without subsequent operation.

3. Laparatomy is to be recommended not only in cases before rupture, and after rupture for the purpose of stopping hemorrhage which threatens life, but also after bleeding has ceased, and even if hematocele has formed, because by it we remove all present and future danger, and insure our patient a very rapid and uninterrupted convalescence. Laparatomy has proven to be, if performed by skilful hands and with all necessary precautions, a perfectly safe operation.

The remaining tube should not be removed—*i. e.*, the tube not the seat of fetation—as has been recommended by some to prevent a recurrence of pregnancy in that tube, unless it is markedly diseased. Removal of it became necessary only once in the four cases operated on for ectopic gestation by him. Two cases subsequently became pregnant, one has been delivered of two living children, and another is now in her seventh month of pregnancy.

Dr. Florian Krug, of New York, gave a short historical sketch of the employment of

TRENDELENBURG'S POSTURE IN GYNECOLOGY.

Dr. Krug witnessed a suprapubic cystotomy done in this position by Dr. Willy Meyer, formerly Prof. Trendelenburg's assistant. He was immediately impressed with the great advantages this method offered for abdominal work, and at once set out to make use of it. He has since done over one hundred and fifty laparatomies in this posture, and has induced a great many operators on this side of the Atlantic to adopt it.

Dr. Krug claimed the following advantages for the method : If the patient's pelvis is raised up so that the symphysis forms the highest point and the body comes on an incline of at least 45° to the horizontal, all the viscera of the abdominal cavity will gravitate toward the diaphragm and the pelvis become free and easy of access. The small intestines will hardly come into view and never trouble the operator during the operation. The operator is enabled to see everything that he is doing and need not grope around in the dark. All bleeding points are readily detected and tied. In weak and anemic patients the posture is a great advantage, preventing shock from acute anemia of the brain. In all his operations he had never found any objectionable point or disadvantage in this posture. There are different ways of putting the patient in this position, very simple and very complicated ones. In most of his operations he had used the head-rest of an operating table, to which cushions were fastened with straps. Trendelenburg himself has had a very complicated operating chair constructed, which answers all requirements. Several new devices have been brought out in New York lately.

Dr. Krug had lately constructed a frame of galvanized iron, which can be screwed to any laundry or operating table. The upper part of this frame is covered with sailing canvas, a material which is durable, easily sterilized, and cheap. Straps are provided for the knees and ankles of the patient, whose pelvis can, by a simple mechanical arrangement, be elevated to an angle of 45° to 60°, and lowered again if required. The frame can easily be carried or taken along in a street car, and can be used on any kind of a table; it is easily cleaned and sterilized, and it is cheap.

Dr. R. T. MORRIS, of New York, said that those who had once seen an operation in this position would appreciate fully the great value of Trendelenburg's invention.

Dr. H. O. MARCY, of Boston, presented photographs showing a modification of the Trendelenburg chair.

Dr. J. H. CARSTENS, of Detroit, spoke of the danger of atmospheric infection from the air rushing into the abdominal cavity, as had been stated was the case as soon as the abdominal walls were opened. He considered that that would be a decided objection to the Trendelenburg position.

Dr. WILLY MEYER, of New York, replied to Dr. Carstens's objection that the danger from atmospheric infection would be very slight, certainly no greater than in amputations and similar open work, in which cases he always expected union by first intention. He then gave a demonstration of the Trendelenburg posture on a table after Trendelenburg's original model that he had imported.

Dr. KRUG, in closing the discussion, said that ever since he had used the Trendelenburg posture he wondered how he ever got along before. He considered that it would be a very fine distinction between the amount of air which entered the abdominal cavity in this posture and that which enters in ordinary operations. He had used the operation in from one hundred and fifty to two hundred laparatomies, and was willing to match his results with those of anybody else who operates in a horizontal position.

Dr. RUFUS B. HALL, of Cincinnati, read an essay upon

### SUPPURATING CYSTS DEVELOPED FROM ADHERENT OVARIES AFTER REPEATED ATTACKS OF INFLAMMATION; AND SECONDARY OPERATIONS FOR REMOVAL OF DOUBLE INTRA-LIGAMENTOUS CYSTS.

His conclusions were to the effect that, if these cases were operated upon early, just as soon as the physician was certain that nothing but an operation could bring the hoped for relief, the operator would not be called upon to treat such desperate cases as those reported. No operator is justified in leaving an abdominal operation incomplete, except in malignant disease, for the reason that all other growths can be removed, and it should be done when once attempted. As long as the general practitioner persists in pursuing what he pleases to call conservative treatment in these cases, and keeps the patients under his care just as long as he can keep breath in them, and surgeons of the older class turn these patients from their consulting rooms as non-operative cases and thus defer it, or the cases made still more complicated by incomplete operations, men engaged in this special work will continue to see just such desperate cases. While this state of affairs exists, what can we hope for other than a high mortality in these delayed cases, and who should be held responsible for the deaths ?

Dr. M. Rosenwasser, of Cleveland, wished to protest against the assertion that these intestinal adhesions were due to the first operation. Intestinal adhesions are common to the broad ligament, and not to the cyst, and will be found there whether you operate the first time or the second time. He also wished to contradict the assertion that these cysts ought to be removed. It is better occasionally not to attempt removal. In some cases it is much better to stitch the cyst to the abdominal wall and drain, leaving the cyst wall alone.

Dr. H. O. Marcy, of Boston, said that in a certain class of cases he had felt that we must stitch and drain. In another class of cases you can readily get behind it, close it down by suturing, and close your wound as in a simple operation.

Dr. W. W. Seymour, of Troy, called attention to the method, suggested originally by Hegar, of approaching these collections of pus through the ischio-rectal space. There is the advantage of not invading cavities not ordinarily septic.

Dr. Hall, closing the discussion, stated that the first case illustrated very forcibly the advisability of an operation on these cases of repeated attacks of inflammation early,—as early as it is found that nothing but an operation can cure them. These cases further illustrate what Dr. Price has said, that we get the worst class of cases among the best class of patients, those of refinement and culture.

### Third Day—Afternoon Session.

Dr. Charles A. L. Reed, of Cincinnati, presented

### OBSERVATIONS ON THE SURGICAL MANAGEMENT OF PELVIC ABSCESS.

Pelvic abscess implies an accumulation of pus within the pelvis, but outside of the uterine appendages. Pus within the appendages is known either as pyosalpinx or ovarian abscess, as the case may be. The terms are not interconvertible. The old pathology, which represented every accumulation of pus within the pelvis as cellular in its origin and location, was pernicious ; but to deny the existence of such cases is wide of the truth, and to treat all intrapelvic pus cases as tubal or ovarian is likewise pernicious. Three illustrative cases were given in which the abdomen had been opened for pus tubes, but the appendages found free from disease. The pus was found within the cellular tissue and broad ligament, and was evacuated by incision along Poupart's ligament. The suggestion for surgical management consists in adopting this operation as a line of practice. By this means we are enabled to treat the appendages, if diseased, and we are enabled to place the field of operation under complete control.

Dr. Paul F. Mundé, of New York, present by invitation, said that he felt diffident about rising to this question, although he had just said to Dr. Reed that it was a subject he felt very strongly upon and one which he had had considerable experience with. He quite agreed with Dr. Reed. There are many gentlemen who believe that laparatomy is the only thing. There are many who maintain that there is no such thing as pelvic abscess—abscess of the pelvic cellular tissue ; everything must come from the tubes ; everything is necessarily a pyosalpinx, and could only get into the pelvic tissues secondarily. He did not believe anything of the kind. He knew there were gentlemen who believed it. From his own experience he could see no reason why there should not be just as much plastic exudation between the layers of the broad ligament, or wherever there is cellular tissue in the pelvis, as in any other part of the body where there is cellular tissue. We have boils and abscesses in other parts of the body, and why should we not have them in the pelvis ? Besides, we know we have effusions of blood in the pelvis. We know also that ovarian tumors and fibroid tumors develop in the broad ligament, and dissect up the peritoneum almost as far as the diaphragm. Why should plastic material from the blood not be exuded between the layers of the broad ligament and into the cellular tissue? That granted, why should it not break down and become pus? Lawson Tait has taught us to recognize these conditions of pus in the peritoneal cavity by bimanual examination without opening the abdominal cavity. The differential diagnosis is not always an easy one to make. Whenever a collection of plastic lymph in the pelvic cavity is immovable, it is usually extraperitoneal, and the same would apply to a fluctuating mass containing either pus or blood; when there is a limited movability to it or a limited movability to the uterus, particularly when pulled downward or upward, it was intraperitoneal.

Dr. Joseph Price, of Philadelphia, presented

A PLEA FOR EARLY HYSTERECTOMY AND PUERPERAL HYSTERECTOMY.

The history of abdominal and pelvic surgery is all aglow with the heroic effort, the personal sacrifices of its pioneers. Surgical invention has greatly improved upon and simplified methods. Where the spirit of innovation has bungled, the better genius of surgery has corrected. We are having cleanliness without the aid of chemical irritants and disinfectants. We are rapidly advancing to accept early operation as a dictum in pelvic and abdominal surgery. I can find no delight in so-called conservative methods. My experience disproves and condemns them. It will become an axiom of surgery not to delay longer than to establish the fact that operation will be necessary at some time. This granted, the earlier such operation is done the fewer will be the complications, and all the dangers attending operation will be diminished or avoided. There will be a shorter operation, less handling of the parts, less shock, surgical and dynamic, and quicker convalescence.

The simple entering the abdomen is without danger in the hands of experienced men. Now, when surgical experience proves that the simpler the operation the less dangerous it is, and that the danger increases by exact gradation as the complications increase, what other conclusion to the argument is there than to demand early operation for conditions that in almost all cases eventuate seriously? This is especially true in fibroid tumors. The removal of the appendages is proven to be efficient, in a majority of cases, in controlling hemorrhage, just as it is the clinical testimony that in almost all cases of fibroid disease there is real disease of the ovary itself. In large tumors the ugly nature of the complications, combined with the gradually increasing discomfort, is such that makes delay criminal. We must operate before the patient is past help, if we would save her. Surgery as a last resort after temporizing has failed is no criterion of what surgery can accomplish, and is no measure or standard by which it may be judged.

At an executive session the following officers were elected: *President*, Dr. A. Vander Veer, of Albany; *Vice-Presidents*, Dr.

H. E. Hill, Saco, Me., and Dr. R. T. Morris, New York ; *Secretary*, Dr. William Warren Potter, Buffalo ; *Treasurer*, Dr. X. O. Werder, Pittsburg ; *Executive Council*, Drs. C. A. L. Reed, Cincinnati ; Lewis S. McMurtry, Louisville ; George H. Rohé, Baltimore ; James F. W. Ross, Toronto ; and William W. Seymour, Troy.

The following-named physicians were elected to Fellowship :

*Honorary*—Dr. George Jackson Fisher, Sing Sing, N. Y. ; Dr. Gratz Ashe Moses, St. Louis (transferred from Ordinary Fellow) ; Dr. Juan M. Rodriguez, City of Mexico ; Dr. Juan Santos Fernandez, Havana, and Dr. E. Pietranera, Cordova, Argentine Republic.

*Corresponding*—Dr. Henry S. Griffin, Hamilton, Ont., Canada, and Dr. Henry T. Machell, Toronto, Ont., Canada.

*Ordinary*—Dr. I. H. Cameron, Toronto, Ont., Canada ; Dr. Henry Gibbons, Jr., San Francisco, Cal. ; Dr. Frances L. Haynes, Los Angeles, Cal. ; Dr. John R. Haynes, Los Angeles, Cal. ; Dr. J. B. S. Holmes, Rome, Ga. ; Dr. Henry Howitt, Guelph, Ont., Canada ; Dr. George Ben Johnston, Richmond, Va. ; Dr. Willis G. Macdonald, Albany, N. Y. ; Dr. James McCann, Pittsburg, Pa.; Dr. R. Barrington Nevitt, Toronto, Ont., Canada; Dr. George S. Peck, Youngstown, O. ; Dr. Edmund M. Pond, Rutland, Vt. ; Dr. William Porter, Jr., Hartford, Conn. ; Dr. E. Arnold Praeger, Nanaimo, B. C. ; Dr. Charles N. Smith, Toledo, O. ; Dr. Edwin Walker, Evansville, Ind.

St. Louis was selected as the next place of meeting and the date fixed upon was the 3d Tuesday of September, 1892.

